# Long-term survival in patients with pancreatic cancer treated with second-line liposomal irinotecan plus 5-fluorouracil/leucovorin: observations from Korea, Italy, and Germany

**DOI:** 10.1016/j.esmogo.2025.100217

**Published:** 2025-08-13

**Authors:** S. Lonardi, K. Potthoff, L. Procaccio, C. Yoo, T. Macarulla, F. Hedouin-Biville, G.W. Prager

**Affiliations:** 1 Veneto Institute of Oncology IOV – IRCCS, Padova, Italy; 2 iOMEDICO AG, Freiburg, Germany; 3 ASAN Medical Center, University of Ulsan College of Medicine, Seoul, Korea; 4 Vall d’Hebron University Hospital and Vall d’Hebron Institute of Oncology, Barcelona, Spain; 5 Servier Affaires Médicales, Suresnes, France; 6 Medical University of Vienna, Department of Medicine I, Vienna, Austria

**Keywords:** pancreatic cancer, irinotecan, 5-fluouracil, leucovorin, survival

## Abstract

Pancreatic cancer (PAC) is an aggressive disease with poor clinical outcomes. Liposomal irinotecan in combination with 5-fluorouracil and leucovorin (nal-IRI+5-FU/LV) is the only approved therapy for metastatic PAC following gemcitabine-based therapy, based on the survival benefit demonstrated in the phase III NAPOLI-1 trial. Factors associated with long-term survival in this trial included age ≤65 years, Karnofsky performance status (KPS) ≥90, neutrophil-to-lymphocyte (N/L) ratio ≤5, carbohydrate antigen (CA) 19-9 <59-times the upper limit of normal (ULN), and no liver metastases. Using real-world data from studies conducted in Korea, Italy, and Germany, this review aims to assess the suitability of prognostic factors identified in the NAPOLI-1 trial nomogram. In these real-world studies, a high CA19-9 level and a low N/L ratio were associated with long-term survival in patients treated with nal-IRI+5-FU/LV. The impact of albumin levels, body mass index (BMI), liver metastasis, and KPS on survival identified from the NAPOLI-1 trial was confirmed in some real-world analyses but not consistently. Factors such as patient age and number of previous lines of treatment that were not identified in the NAPOLI-1 nomogram may be associated with long-term survival with nal-IRI+5-FU/LV in the real-world. In conclusion, this review has shown that while prognostic factors are useful for patient stratification, their predictive value on the efficacy of nal-IRI+5-FU/LV is low, thus this treatment may also result in long-term survival in patients with apparently unfavorable characteristics.

## Highlights

• nal-IRI+5-FU/LV is an approved treatment of metastatic pancreatic cancer (mPAC) following gemcitabine-based therapy.
• NAPOLI-1 investigated nal-IRI+5-FU/LV in mPAC and factors associated with long-term survival were identified in a nomogram.
• This review assesses the impact of these prognostic factors in the real-world in Korea, Italy and Germany.
• A high CA19-9 level and a low N/L ratio were associated with long-term survival in the real-world.
• Other factors, such as albumin levels and BMI, were confirmed in some of the real-world studies, but not consistently.

## Introduction

Pancreatic cancer (PAC) is an aggressive disease with a poor prognosis, with ∼85% of patients presenting with locally advanced PAC or metastatic PAC (mPAC) at diagnosis.^1^ Despite treatment, only 10% of patients with PAC survive for >5 years.^2^ Survival rate is further reduced in patients with mPAC, with <25% of patients surviving for >1 year and only 2% of patients surviving for >5 years.^3^

The recommended first-line (1L) treatment of patients with mPAC and an Eastern Cooperative Oncology Group (ECOG) performance status of 0 or 1 is infusional 5-fluorourcacil (5-FU), leucovorin (LV), irinotecan and oxaliplatin (FOLFIRINOX), or gemcitabine combined with albumin-based paclitaxel.^4^ Liposomal irinotecan in combination with 5-FU and LV (nal-IRI+5-FU/LV) is the only approved second-line (2L) therapy for mPAC following gemcitabine-based therapy.^4^ Moreover, based on the efficacy shown in the NAPOLI-3 trial, liposomal irinotecan in a nal-IRI+5-FU/LV + oxaliplatin (NALIRIFOX) regimen has also recently been approved as a 1L therapy for mPAC.^5^ No specific therapies are recommended for third-line (3L) treatment of mPAC due to a lack of treatments with solid evidence of efficacy and the poor nutritional status and performance status of most patients in the 3L. If patients have good performance status, inclusion in a clinical trial of 3L treatment should be considered, while best supportive care is recommended in all other cases.^4^

The 2L recommendation for the treatment of mPAC stems from the pivotal global phase 3 NAPOLI-1 trial which reported a 1-year overall survival (OS) rate of 26% [95% confidence interval (CI) 18% to 35%] in patients with mPAC treated with nal-IRI+5-FU/LV, having previously received gemcitabine-based therapy.^6^ The estimated probability of survival at 1-year in NAPOLI-1 is in line with the 1-year OS of 10% to 23% reported in a large European systematic review of observational studies which encompassed all mPAC stages and lines of therapy.^7^ Despite differences in baseline patient characteristics, a retrospective observational database study of >280 cancer clinics (699 patients) in the USA showed a similar 1-year OS rate of 29.1% (95% CI 24.0% to 34.3%) in those who received at least four treatment cycles of nal-IRI-based regimens in any line.^8^

In NAPOLI-1, factors associated with long-term survival (≥1 year) in patients receiving nal-IRI+5-FU/LV were age ≤65 years, Karnofsky performance status (KPS) ≥90, neutrophil-to-lymphocyte (N/L) ratio ≤5, carbohydrate antigen (CA) 19-9 <59-times the upper limit of normal (ULN), and no liver metastases. In an attempt to predict long-term survival in patients treated with nal-IRI+5-FU/LV, a nomogram was derived from NAPOLI-1, which included baseline KPS, albumin level, N/L ratio, liver metastases, CA19-9, disease stage at diagnosis, body mass index (BMI), and treatment arm (nal-IRI+5-FU/LV).^9^ However, a review of real-world data is needed to determine whether these characteristics are predictive of response to nal-IRI+5-FU/LV following gemcitabine-based therapy, outside of the strict confines of a clinical trial. The identification of prognostic characteristics of response to nal-IRI+5-FU/LV treatment would improve patient selection and survival outcomes.

This review assesses the suitability of prognostic factors identified in the NAPOLI-1 predictive nomogram using real-world data from studies conducted in Korea, Italy, and Germany, in patients with mPAC achieving long-term survival with nal-IRI+5-FU/LV treatment, following gemcitabine-based therapy. Collated patient characteristics from these real-world studies are summarized in Table 1.

**Table 1:** Characteristics of patients with metastatic pancreatic cancer in each study included within this narrative review

	Korean pooled analysis		Italian real-world analysis^12^, ^13^, ^14^	German iOMEDICO registry^15^
	Yoo et al.^10^	Park et al.^11^		
Sample size, (*n*)	86	104	296	101^a^
Median age (range), years	61 (37-79)	64 (35-78)	64.4 (30.1-82.7)	70.6 (61.6-76.1)
Male (%)	60.5	58.7	50.3	51.5^b^
ECOG PS 0-1 (%)	100.0	82.7	44.3^c^	92.1^b^
Receiving nal-IRI+5-FU/LV in 2L (%)	31.4	100.0	72.3	100.0

1L, first-line treatment; 2L, second-line treatment; 5-FU, 5-fluorouracil; ECOG PS, Eastern Cooperative Oncology Group performance status; IRI, irinotecan; LV, leucovorin.

a Metastatic pancreatic cancer at start of 1L treatment.

b At start of 1L treatment.

c ECOG PS 0 reported.

## Summary of studies included in this review

### Korean pooled analysis

Data were pooled from two studies conducted by the Korean Cancer Study Group. Both studies were retrospective, multicenter, open-label, observational analyses.^10^^,^^11^ The methodology of these two studies has been described in detail previously.^10^^,^^11^

This pooled analysis included patients with mPAC treated with nal-IRI+5-FU/LV after treatment with gemcitabine (*n* = 190), of which 127 (66.8%) were treated in 2L. Patients receiving nal-IRI+5-FU/LV in the 2L had a median progression-free survival (PFS) of 3.7 months (95% CI 2.3-5.1 months) and OS of 7.7 months (95% CI 5.6-9.8 months). In this pooled analysis, 24.5% of patients survived long-term (defined as survival of >10 months).

### Italian real-world analysis

This study was a retrospective, multicenter analysis including 296 patients with mPAC who received nal-IRI+5-FU/LV after failure of a gemcitabine-based therapy at 11 Italian centers between June 2016 and November 2018.^12^, ^13^, ^14^ Of the 296 patients included in this study, 214 (72.3%) received nal-IRI+5-FU/LV in the 2L and 77 (26.0%) were defined as long-term survivors (≥12 months). This real-world analysis showed patients with mPAC treated with nal-IRI+5-FU/LV following treatment with gemcitabine had a median PFS of 3.2 months (95% CI 3.0-3.7 months) and OS of 7.2 months (95% CI 5.9-8.7 months).^13^ Long-term survivors in this study had a median OS of 17.5 months (95% CI 16.4-21.1 months) and PFS of 10.3 months (95% CI 9.0-12.2 months).^14^ Several patient characteristics were assessed for their association with survival.

### German iOMEDICO registry

The Tumour Registry Pancreatic Cancer (NCT02089269^15^) study is an ongoing, prospective clinical cohort study that includes patients with locally advanced, inoperable or mPAC at the start of 1L treatment from 128 centers across Germany.^15^ Recruitment to this study was paused in April 2022 and data reported here are using a database cut-off of 30 June 2024.

Of the 2162 patients recruited, 208 patients (9.6%) with locally advanced PAC had received 1L treatment, of which 92 (44.2%) had received 2L treatment. Of the recruited patients, 1954 (90.4%) patients had mPAC and 857 (43.9%) of these patients had received 2L treatment. Of the patients receiving 2L treatment, 7 (7.6%) patients with locally advanced PAC and 101 (11.8%) patients with mPAC received nal-IRI+5-FU/LV in the 2L, following 1L gemcitabine. Due to the small number of patients with locally advanced PAC patients, these were excluded from the current analysis.

Of the patients with mPAC receiving 2L nal-IRI+5-FU/LV post-gemcitabine therapy, 18 (17.8%) were defined as long-term survivors (≥12 months) and 63 (62.2%) as short-term survivors (<12 months). However, 20 (19.8%) patients could not be categorized as long- or short-term survivors as they were observed for <12 months after the start of 2L treatment at the database cut-off.

This real-world analysis showed patients with mPAC treated with nal-IRI+5-FU/LV following treatment with gemcitabine had a median PFS of 3.5 months (95% CI 2.8-4.3 months) and OS of 6.6 months (95% CI 5.1-9.0 months), respectively. Long-term survivors in this study had a median PFS of 11.4 months (95% CI 4.3-15.7 months), compared with a median PFS of 2.6 months (95% CI 1.9-3.4 months) for short-term survivors. The median OS of long-term survivors was 17.6 months (95% CI 14.2 to NA months), compared with a median OS of 4.3 months (95% CI 2.8-5.3 months) for short-term survivors.

### Patient populations included in this review

Data from patients with locally advanced or mPAC who had received prior gemcitabine-based therapy were utilized from the studies discussed in this review. Where possible, patient populations were selected to be consistent across studies. However, there were some differences in patient populations and definitions of long-term survival across these studies:

• The inclusion criteria for the German study did not specify nal-IRI+5-FU/LV, and instead patients were included at the start of their palliative 1L treatment. So, for this review, data from patients treated with nal-IRI+5-FU/LV in the 2L following gemcitabine-based therapy were extracted from the German study to ensure a similar population for comparison with the Korean and Italian studies.
• The definition of long-term survival used across these real-world studies differs. The Italian and German studies defined long-term survival as ≥12 months,^12^, ^13^, ^14^, ^15^ which is consistent with the definition used in NAPOLI-1.^6^ However, the Korean pooled analysis defined long-term survival as >10 months.^10^^,^^11^

Despite the difference in long-term survival definitions, the Korean pooled analysis definition of long-term survival will still capture patients that survived ≥12 months. Despite the 12-month cut-off for long-term survival used in the NAPOLI-1 trial,^6^ there is no clearly defined, universal definition of long-term survival in PAC. Therefore, the 10-month definition used in the Korean pooled analysis may be equally useful to assess prognostic factors, even if this limits comparison between data from the Korean pooled analysis and other studies included in this review.

## Factors associated with long-term survival in the real world

### Previously identified factors from the NAPOLI-1 nomogram

#### Albumin level

The predictive nomogram derived from NAPOLI-1 showed that patients treated with nal-IRI+5-FU/LV following gemcitabine therapy with an albumin level ≥4 g/dl survived longer than patients with an albumin level of <4 g/dl (hazard ratio [HR] 0.67, 95% CI 0.53-0.85, *P* = 0.0008).^9^ Analyses of real-world studies appear to provide some support for high albumin levels as a reliable predictor of long-term survival in patients with mPAC treated with nal-IRI+5-FU/LV following gemcitabine therapy, but data are mixed.

In the Korean pooled analysis, albumin levels were not included in the analysis,^10^^,^^11^ but in one of the studies, multivariate analysis identified albumin level ≥3.5 g/dl as an independent prognostic factor for prolonged OS (HR 0.42, 95% CI 0.30-0.59).^11^ In the Italian real-world analysis, despite the interim analysis suggesting an impact of albumin levels ≥4 g/dl on survival consistent with the NAPOLI-1-derived nomogram,^12^ the final analysis did not confirm an association between albumin levels ≥4 g/dl and longer OS.^13^ Cases of long-term survivors receiving nal-IRI+5-FU/LV in the 2L have been reported in patients with an albumin level ≥4 g/dl (but only just over the threshold at 4.6 g/dl),^16^ but also in patients with albumin levels of <4 g/dl.^17^

#### Neutrophil-to-lymphocyte ratio

Univariate analyses of NAPOLI-1 data showed that a N/L ratio of ≤5 was significantly associated with longer OS in patients treated with nal-IRI+5-FU/LV, following treatment with gemcitabine (HR 0.58, 95% CI 0.46-0.74, *P* < 0.0001).^9^ The association between N/L ratio and long-term survival in patients treated with nal-IRI+5-FU/LV following treatment with gemcitabine therapy has also been supported in the real-world studies. In the Italian real-world analysis, a N/L ratio of ≤5 was associated with long-term survival (odds ratio [OR] 9.74, 95% CI 2.12-74.22, *P* = 0.0093; Table 2).^13^^,^^14^ An isolated case of long-term survival of PAC patients treated with nal-IRI+5-FU/LV with a N/L ratio of >5 has been reported.^17^

**Table 2:** Characteristics associated with survival outcomes in multivariate analyses in the Italian real-world analysis^12^, ^13^, ^14^

Characteristic	Comparison	OR (95% CI)	*P* value
Age, years (ref: ≥70)	<70	0.32 (0.14-0.70)	0.0052
Primary tumor location (ref: Head/uncinated process)	Other	0.31 (0.13-0.68)	0.0048
AJCC tumor grading (ref: G3-G4)	GX	3.53 (1.23-11.26)	0.0243
	G1-G2	3.74 (1.31-11.46)	0.0161
Liver metastasis at start of nal-IRI+5-FU/LV therapy (ref: Yes)	No	2.98 (1.37-6.59)	0.0061
Previous anti-cancer therapy for non-metastatic disease: adjuvant (ref: No)	Yes	3.55 (1.31-9.99)	0.0140
Previous anti-cancer therapy for non-metastatic disease: neo-adjuvant (ref: No)	Yes	4.84 (1.30-19.19)	0.0200
Previous lines of therapy for metastatic disease (ref: ≥1)	0	5.87 (0.86-56.02)	0.0829
Biliary stenting at any time (ref: Yes)	No	2.66 (1.11-6.76)	0.0326
Baseline ECOG PS (ref: ≥1)	0	2.72 (1.29-5.89)	0.00933
Baseline CA19-9 (ref: >37 ng/ml)	≤37	2.46 (0.98-6.19)	0.0546
Neutrophil-to-lymphocyte ratio (ref: >5)	≤5	9.74 (2.12-74.22)	0.00933
Platelets (ref: ≤150 × 10^9^/l)	>150	3.31 (1.23-10.42)	0.0258
Hemoglobin (ref: <11 g/dl)	≥11	2.79 (1.21-7.00)	0.0211

AJCC, American Joint Committee on Cancer; CA, carbohydrate antigen; CI, confidence interval; ECOG PS, Eastern Cooperative Oncology Group performance status; OR, odds ratio; ref, reference.

#### CA19-9 level

The nomogram derived from NAPOLI-1 trial showed that a CA19-9 >41-times the ULN (>1542 U/ml) was associated with poorer survival (HR 1.62, 95% CI 1.29-2.03, *P* < 0.0001) in patients treated with nal-IRI+5-FU/LV in the 2L.^9^ In the Korean pooled analysis, there was no significant difference in CA19-9 levels >2-times the ULN between long-term (>10 months) and short-term survivors (<10 months; Table 3).^10^^,^^11^ However, in one of the studies, multivariate analysis identified CA19-9 >ULN (≥40 U/ml) was an indicator for shorter PFS (HR 1.47, 95% CI 1.07-2.03, *P* = 0.018) and OS (HR 1.79, 95% CI 1.21-2.64, *P* = 0.003).^11^ In the Italian real-world analysis, normal levels of CA19-9 (≤37 U/ml) were associated with long-term survival (OR 2.46, 95% CI 0.98-6.19, *P* = 0.0546; Table 2).^13^^,^^14^

**Table 3:** Patient characteristics associated with >10 months survival in the Korean pooled analysis^a^^,^^10^^,^^11^

Characteristic	Patient subgroup		*P* value
	OS <10 months (*n* = 83), *n* (%)	OS >10 months (*n* = 27), *n* (%)	
Age, years			
<65	55 (63.3)	13 (48.1)	0.113
≥65	28 (33.7)	14 (51.9)	
Sex			
Male	44 (53.0)	13 (48.1)	0.825
Female	39 (47.0)	14 (51.9)	
Prior surgery	14 (30.4)	8 (44.4)	0.382
Liver metastasis	61 (73.5)	12 (44.4)	0.009^a^
Lung metastasis	16 (19.3)	9 (33.3)	0.185
Peritoneal metastasis	33 (39.8)	8 (29.6)	0.371
CA 19-9 >2 × ULN	50 (78.1)	16 (66.7)	0.281

CA, carbohydrate antigen; OS, overall survival; ULN, upper limit of normal.

a 80 patients who survived at the time of analysis and follow-up duration <10 months were excluded in this analysis.

#### Body mass index

The nomogram derived from NAPOLI-1 showed that patients with a BMI of >25 kg/m^2^ survived for longer than patients with a BMI ≤25 kg/m^2^, indicating BMI as a prognostic factor for long-term survival in patients treated with nal-IRI+5-FU/LV.^9^ In the German iOMEDICO registry, patients surviving for <12 months had a median (25% to 75% quartile range) BMI of 23.2 (20.0-27.5 BMI), whereas long-term survivors (≥12 months) had a median (25% to 75% quartile range) BMI of 22.5 (21.5-25.2 BMI; Table 4) at the start of their 1L treatment, but the difference is too small to draw any conclusion.^15^ In the real-world study in Italy, BMI was not associated with long-term survival, but a lower cut-off (18.5 kg/m^2^) was used.^13^

**Table 4:** Patient and tumor characteristics of short- (<12 months) and long-term (≥12 months) survivors following 2L nal-IRI+5-FU/LV treatment in the German Registry^15^

	Short-term survivors (*n* = 63)	Long-term survivors (*n* = 18)
Age^a^, years		
<70, *n* (%)	34 (54.0)	7 (38.9)
≥70, *n* (%)	29 (46.0)	11 (61.1)
BMI^a^, median (25%/75% quantiles)	23.2 (20.0-27.5)	22.5 (21.5-25.2)
Charlson comorbidity index, *n* (%)		
0	51 (81.0)	12 (66.7)
≥1	12 (19.0)	6 (33.3)
ECOG PS^a^, *n* (%)		
0	27 (42.9)	8 (44.4)
1	32 (50.8)	8 (44.4)
≥2	4 (6.3)	2 (11.1)
Metastatic site at 2L, *n* (%)		
Liver	46 (73.0)	10 (55.6)
Lung	18 (28.6)	9 (50.0)
Peritoneum	18 (28.6)	6 (33.3)
Lymph nodes	27 (42.9)	5 (27.8)
Pleura	1 (1.6)	0 (0.0)
Other	17 (27.0)	6 (33.3)
Missing	0 (0.0)	0 (0.0)

2L, second-line therapy; ECOG PS, Eastern Cooperative Oncology Group performance status; SD, standard deviation.

a At start of first-line treatment.

There has been a report of long-term survival in patients treated with nal-IRI+5-FU/LV in the 2L who have a BMI of >25 kg/m^2^, which corroborates the use of BMI as a prognostic factor for long-term survival with the treatment of nal-IRI+5-FU/LV.^18^ However, several reports have demonstrated that long-term survival with nal-IRI+5-FU/LV treatment is possible in patients with a BMI of ≤25 kg/m^2^.^16^^,^^17^^,^^19^

#### Absence of liver metastases

The NAPOLI-1 nomogram showed that an absence of liver metastases was associated with longer survival in patients treated with nal-IRI+5-FU/LV (HR 0.58, 95% CI 0.45-0.75, *P* < 0.0001).^9^ Consistent with the NAPOLI-1-derived analyses,^6^^,^^9^ the real-world Korean pooled analysis showed a lower proportion of patients surviving >10 months (44.4%) had liver metastases compared with patients with an OS of <10 months (73.5%; *P* = 0.009; Table 3).^10^^,^^11^ Similar data came from the German iOMEDICO registry, which reported that 55.6% of long-term survivors (≥12 months) had liver metastases, compared with 73.0% of short-term survivors (<12 months; Table 4).^15^ These data were also confirmed in the Italian study, which showed that the absence of liver metastases at the start of nal-IRI+5-FU/LV therapy was associated with long-term survival (OR 2.98, 95% CI 1.37-6.59, *P* = 0.0061; Table 2).^13^^,^^14^

Generally, these real-world studies confirmed a lower incidence of liver metastases in the long-term survivors’ groups compared with the short-term survivors, even if globally the presence of liver disease was higher than among the NAPOLI-1 trial participants. The negative prognosis associated with liver metastases is well known; however, the predictive impact on nal-IRI+5-FU/LV efficacy is unclear as long-term survival of patients treated with 2L nal-IRI+5-FU/LV has been reported, despite the presence liver metastases in a number of cases.^17^, ^18^, ^19^ This highlights that while the absence of liver metastases is a predictive factor for longer survival in patients, long-term survival is possible in patients with liver metastases when treated with nal-IRI+5-FU/LV. However, further work with a larger sample size of long-term survivors would be needed to elucidate whether the presence of liver metastases is useful as a predictive tool in the real-world.

#### Patient performance status

As previously reported in the NAPOLI-1 nomogram, a KPS of ≥90 is associated with long-term survival in patients treated with nal-IRI/5-FU/LV.^9^ A KPS of ≥90 is equivalent to an ECOG performance status of 0.^20^ In the German registry study, 44.4% of patients who survived long-term (≥12 months) and 42.9% of patients who survived short-term (<12 months) had an ECOG performance status of 0 at the beginning of 1L treatment.^15^ The Italian real-world analysis showed a significantly shorter OS in patients with an ECOG performance status of ≥1 than those with an ECOG performance status of 0 (OR 2.72, 95% CI 1.29-5.89, *P* = 0.00933; Table 2).^14^ A higher proportion of patients included in this real-world analysis (56%) had an ECOG performance status of ≥1 than in the NAPOLI-1 trial (41%).^6^^,^^12^, ^13^, ^14^

#### Tumor stage

The NAPOLI-1 nomogram indicated that patients who presented with PAC staged (TNM [AJCC 2009, 7th version] classification) lower than 4 at diagnosis survived for longer than patients diagnosed with stage 4 PAC.^9^ There are limited data on the effect of tumor stage on survival in patients treated with nal-IRI+5-FU/LV in the real-world analyses included in this review, so these cannot be used to assess the suitability of this metric to predict survival.^10^, ^11^, ^12^^,^^14^^,^^15^

#### Summary

The identification of predictive factors for long-term survival in the NAPOLI-1 nomogram could be more relevant to the confines of a randomized clinical trial. Real-world data have shown that long-term survival with nal-IRI+5-FU/LV post-gemcitabine is not always associated with the factors identified from NAPOLI-1. However, a high CA19-9 level and a low N/L ratio were associated with long-term survival in patients treated with nal-IRI+5-FU/LV in clinical trials and confirmed in real-world studies, but the threshold of CA19-9 levels that may be associated with long-term survival is not clear and provides an avenue for further investigation.

The impact of albumin levels, BMI, liver metastasis and performance status on survival that were identified from the NAPOLI-1 trial was confirmed in some real-world analyses but not consistently.

### Other factors not included in the NAPOLI-1 nomogram

Other prognostic factors associated with long-term survival in patients treated with 2L nal-IRI+5-FU/LV, that were not identified in the NAPOLI-1 trial nomogram, have been identified from these real-world studies. A large proportion of both the Korean pooled analysis and the German registry long-term survivor populations consisted of older (≥70 years of age) patients (51.9% and 61.1%, respectively; Tables 3 and 4). Surprisingly, in the Italian study, an age of ≥70 years was associated with long-term survival in patients receiving nal-IRI+5-FU/LV (Table 2).^12^^,^^14^ Although age was not identified as predictive factor in the nomogram, a patient age of ≤65 years was highlighted as a baseline characteristic associated with long-term survival in the NAPOLI-1 trial, which differs from the association between older age and long-term survival found in the real-world.^6^^,^^9^

In the Italian study, the association between the number of previous treatment lines on long-term survival showed no significant difference between patients treated with ≥1 lines of treatment and patients treated with no lines of treatment of metastatic disease (Table 2).^14^ In one of the Korean studies, the number of lines of previous palliative chemotherapy was not associated with a significant change in median OS or PFS (*P* = 0.64 and *P* = 0.09, respectively).^10^ In the German registry study, all patients had received one previous line of treatment, so the association between the number of treatment lines and long-term survival was not assessed.^15^

Other factors in the Italian study that were associated with long-term survival in patients receiving nal-IRI+5-FU/LV post-gemcitabine included normal platelet count, hemoglobin (≥11 g/dl) level, and an absence of biliary stenting at any time.

## Future directions

This review indicates that real-world evidence should be utilized in the development of updated nomograms from clinical trials to help ensure that prognostic factors identified are consistent across clinical trials and in the real world. The characteristics discussed in this review have also been identified as prognostic factors for long-term survival in patients with PAC. Studies have shown that hypoalbuminemia (defined as <3.6 g/dl), low albumin levels or CA19-9 levels, and ECOG performance status of >1were associated with a shorter OS in patients with PAC.^21^, ^22^, ^23^ Likewise, patients with PAC that are overweight or obese between the ages of 30 and 79 years, or in the year before diagnosis, have reduced OS, irrespective of disease stage and tumor resection status.^24^ More than 50% of patients with PAC present with liver metastases at diagnosis,^25^ and unsurprisingly PAC patients with liver metastases have a poorer prognosis than those with local disease.^26^

Future clinical trials involving the use of nal-IRI+5-FU/LV should be analyzed in a similar way to NAPOLI-1, to gather more information on potential prognostic factors for long-term survival with this treatment. There is a need to gather data from clinical trials, such as NAPOLI-3, along with real-world evidence to assess the use of the factors discussed here to predict the response of patients treated with nal-IRI+5-FU/LV in the 1L. This would allow for future identification of patients that would benefit most from 1L nal-IRI+5-FU/LV treatment based upon these prognostic factors.

Nal-IRI+5-FU/LV is currently recommended in patients with PAC that received gemcitabine-based therapy in the 1L and have an ECOG performance status of 0-1.^4^ Future nomograms and the identification and refinement of prognostic factors could facilitate decision-making and stratify patients with PAC for 2L treatment. Unfortunately, this analysis does not clearly identify characteristics of patients with a high probability of having a long survival with nal-IRI+5-FU/LV. Therefore, this treatment should be considered for all eligible patients that had been pre-treated with gemcitabine, without selecting them based on specific clinical factors.

The identification of more factors could influence future guidelines as to which patients will benefit the most from this regimen. Specifically, the effect of patient age on the likelihood of long-term survival with nal-IRI+5-FU/LV should be explored using both retrospective real-world and clinical trial data. This could provide a clearer understanding of whether patients older or younger than the age of 65 years show increased survival with nal-IRI+5-FU/LV and allow for patient stratification. The survival outcomes of patients receiving nal-IRI+5-FU/LV reported in this review have been obtained in a real-world setting, capturing the heterogeneity of PAC patient populations across countries, age groups, and baseline characteristics. Therefore, the similar PFS and OS values between these studies and NAPOLI-1 provide real-world evidence of the efficacy of nal-IRI+5-FU/LV treatment following gemcitabine-based therapy.

## Conclusions

This review of nal-IRI+5-FU/LV treatment showed that some of the characteristics identified as predictors of long-term survival in the NAPOLI-1-derived nomogram had prognostic value in some of the real-world studies, as described in Table 5. However, most factors included in the NAPOLI-1-derived nomogram were not supported by the real-world analyses included in this review (Table 5). These results indicate the prognostic value of the characteristics identified in the NAPOLI-1-derived nomogram may not be as relevant in the real-world. However, this may reflect the heterogeneity of the studies and patient populations presented in this review. Despite this, the prognostic factors discussed in this review may be of clinical interest for the treatment of patients with PAC and could be utilized to aid clinical decision-making.

**Table 5:** Summary of characteristics associated with long-term survival across real-world studies in this review

	Korean pooled analysis		Italian real-world analysis^12^, ^13^, ^14^	German iOMEDICO registry^15^
	Yoo et al.^10^	Park et al.^11^		
Albumin level	NE	✓	✗	NE
N/L ratio	NE	NE	✓	NE
CA19-9 level	✗	✗	✓	NE
BMI	NE	NE	✗	NE
Absence of liver metastases	✓	✓	✓	✓
Patient performance status	NE	NE	✓	NE
Tumor stage	NE	NE	NE	NE

Ticks (✓) indicate prognostic factors identified in the NAPOLI-1 nomogram that were associated with long-term survival in the respective real-world study. Crosses (✗) indicate prognostic factors identified in the NAPOLI-1 nomogram that were not associated with long-term survival in the respective real-world study.

BMI, body mass index; CA, carbohydrate antigen; N/L, neutrophil-to-lymphocyte; NE, not evaluated.

In conclusion, this study has shown that while prognostic factors are useful for patient stratification, nal-IRI+5-FU/LV may also result in long-term survival in patients with apparently unfavorable characteristics. The prognostic factors discussed in this review, including albumin level, N/L ratio, CA19-9 level, BMI, absence of liver metastases, patient performance status, and tumor stage, have clinical value to stratify patients for treatment with nal-IRI+5-FU/LV and to highlight patients with a favorable prognosis overall. Further work is needed to highlight characteristics that are associated with long-term survival with nal-IRI+5-FU/LV following gemcitabine across both clinical trials and in the real world.
